# Supplementary material for: Assessing the mechanisms of multi-drug resistant non-typhoidal Salmonella (NTS) serovars isolated from layer chicken farms in Nigeria
Source: PLoS One. 2023 Sep 7;18(9):e0290754. doi: 10.1371/journal.pone.0290754 (PMC10484460; doi:10.1371/journal.pone.0290754)
Supplement: S1 File — (DOCX) [file pone.0290754.s001.docx]

Table 1: Number of samples collected, number of farms sampled and prevalence of *Salmonella* per state

| State | No. of farms sampled | No. of samples collected | No. of infected farms (%) | No. of positive samples (%) |
| --- | --- | --- | --- | --- |
| Ogun | 110 | 550 | 72 (65.4) | 135 (24.5) |
| Lagos | 25 | 125 | 14 (56.0) | 19 (15.2) |
| Edo | 18 | 90 | 2 (11.1) | 3 (3.3) |
| Rivers | 22 | 110 | 11 (50.0) | 13 (11.8) |
| Enugu | 38 | 190 | 18 (47.4) | 31 (16.3) |
| Imo | 26 | 130 | 10 (38.5) | 15 (11.5) |
| Gombe | 11 | 55 | 3 (27.3) | 3 (5.5) |
| Bauchi | 21 | 105 | 12 (57.1) | 19 (18.1) |
| Plateau | 74 | 370 | 26 (35.1) | 40 (10.8) |
| Kaduna | 109 | 545 | 33 (30.3) | 55 (10.1) |
| Kano | 45 | 225 | 21 (46.7) | 31(13.8) |
| Katsina | 24 | 120 | 6 (25.0) | 6 (5.0) |
| **Total** | **523** | **2615** | **228** | **370** |
|  |  |  | **43.6% CI_95_ [39.7; 48.3]** | **14.1% IC_95_ [12.8; 15.5]** |

*Fagbamila et al., 2017. doi:10.1371/journal.pone.0173097.t001*

Table 2: Number and type of positive matrices per farm

| **No of positive matrices per farm** | **No of farms (%)** | **Details of the positive matrices** |
| --- | --- | --- |
| 1 | 155 (68) | feed (34); litter (31); faeces (31); dust (30); water (29) |
| 2 | 50 (21.9) | litter-dust (5); litter-faeces (8); litter-feed (5); litter-water (1); dust-faeces (9); dust-feed (4); dust-water (2); faeces-feed (9); faeces-water (3); feed-water (4) |
| 3 | 18 (7.9) | litter-dust-faeces (2); litter-dust-feed (1); litter-dust-water (2); litter-faeces-feed (4); litter-faeces-water (1); litter-feed-water (3); dust-faeces-feed(1); dust-faeces-water (1); dust-feed-water (2); faeces-feed-water (1) |
| 4 | 4 (1.8) | litter-dust-faeces-feed (2); litter-faeces-feed-water (1); dust-faeces-feed-water (1) |
| 5 | 1 (0.4) | litter-dust-faeces-feed-water (1) |

*Extracted from Fagbamila et al., 2017. doi:10.1371/journal.pone.0173097.t002*

Table 3: Number of farms positive for *Salmonella* per each matrix and per state

| State  (No. farm sampled) | N° of farms positive for *Salmonella* | | | | |
| --- | --- | --- | --- | --- | --- |
|  | Litter | Dust | Faeces | Feed | Water |
| Ogun (110) | 24 | 19 | 31 | 25 | 20 |
| Lagos (25) | 4 | 0 | 5 | 4 | 4 |
| Edo (18) | 1 | 1 | 0 | 1 | 0 |
| Rivers (22) | 4 | 5 | 1 | 1 | 1 |
| Enugu (38) | 2 | 5 | 3 | 12 | 4 |
| Imo (26) | 1 | 5 | 2 | 4 | 2 |
| Gombe (11) | 0 | 0 | 2 | 1 | 0 |
| Bauchi (21) | 5 | 5 | 5 | 1 | 2 |
| Plateau (74) | 7 | 5 | 10 | 8 | 6 |
| Kaduna (109) | 13 | 11 | 12 | 9 | 3 |
| Kano (45) | 5 | 7 | 3 | 7 | 5 |
| Katsina (24) | 1 | 0 | 1 | 0 | 4 |
| **Total (523)** | **67** | **63** | **75** | **73** | **52** |
| **Prevalence** | **12.8%** | **12%** | **14.3%** | **13.9%** | **9.7%** |
| **95% Confidence interval** | **[10; 16]** | **[9.4; 15.1]** | **[11.4; 17.6]** | **[11.1; 17.2]** | **[7.3; 12.6]** |

*Extracted from Fagbamila et al., 2017 doi:10.1371/journal.pone.0173097.t003*

Table 4: Species, subspecies and frequency of *Salmonella* serovars isolated.

| S/N | *Salmonella* species | *Salmonella* subspecies | *Salmonella* serovars | Frequency of isolation (%) |
| --- | --- | --- | --- | --- |
| 1 | *S. enterica* | *S. enterica* subspp. *enterica* | *S.* Kentucky | 60 (16.17) |
|  |  |  | *S*. Poona | 21 (5.66) |
|  |  |  | *S. enterica*.subspp *enterica* (inconclusive) | 17 (4.58) |
|  |  |  | *S*. Elisabethville | 15 (4.07) |
|  |  |  | *S*. Larochelle, S. Agama | 14 (3.77) |
|  |  |  | *S.* Saintpaul | 10 (2.70) |
|  |  |  | *S.* Virchow, *S*. Isangi | 9 (2.43) |
|  |  |  | *S*. Give, *S*. Graz, *S.* Cotham, *S.* Liverpool | 8 (2.16) |
|  |  |  | *S.* Muenster, *S.* Telelkebir, *S*. Nigeria, *S*. Jangwani, *S*. Kingston | 7 (1.89) |
|  |  |  | *S.* Hadar, *S.* Weltevreden, *S.* Berlin, | 6 (1.62) |
|  |  |  | *S.* Typhimurium, , *S.* Corvallis, *S.* Canada, *S*. Dugbe | 5 (1.35) |
|  |  |  | *S.* Goldcoast, *S.* Rubislaw, *S.* Chomedey | 4 (1.08) |
|  |  |  | *S.* Namoda, *S.* Ridge, *S.* Vinohrady, *S.* Sanktmark, *S.* Gaminara, *S.* Yaba, *S.* Carno, *S.* Kibi, *S.* Johannesburg, *S.*Schwarzengrund | 3 (0.81) |
|  |  |  | *S.* Offa. *S*. Agbeni, *S.* Hall, *S.* Durham, *S.* Hato *S.* Alachua, *S.* Plymouth, *S,* Chichester, *S.* Madjorio | 2 (0.54) |
|  |  |  | *S.* Bonariensis, *S.* Fresno, *S.* Benfica, *S*. Lattenkamp, *S.* Stanleyville, *S.* Lexington, *S.* Be, *S.* Deversoir, *S.* Enteritidis, *S*. Livingstone, *S*. Lomita, *S*. Gege, *S*. Bradford, *S*. Mapo, *S*. Ealing, *S*. Mbandaka, *S.* Miami, *S*. Sangera, *S*. Millesi, *S*. Kisarawe, *S.* Kande, *S*. Sculcoates, *S*. Herston, *S.* Adelaide, *S.* Dumfries, *S.* Urbana, *S*. Nieukerk, *S*. Kuessel, *S*. Amba, *S*. Dallgow, *S*. Amina, *S*. Muenchen, *S*. Bedford | 1 (0.27) |
|  |  | *S. enterica* subspp. *diarizonae* | S. IIIb: 8: r: z | 1 (0.27) |
| 2. | *S. bongori* |  | S. V: 6, 14: e,n,z15: -; S. V: 48: i:- | 2 (0.54) |

*Extracted from Fagbamila et al., 2017 doi:10.1371/journal.pone.0173097.t004*
