# Supplementary material for: Assessing the mechanisms of multi-drug resistant non-typhoidal Salmonella (NTS) serovars isolated from layer chicken farms in Nigeria
Source: PLoS One. 2023 Sep 7;18(9):e0290754. doi: 10.1371/journal.pone.0290754 (PMC10484460; doi:10.1371/journal.pone.0290754)
Supplement: S2 File — (DOCX) [file pone.0290754.s002.docx]

**Polymerase chain reaction (PCR) Procedures.**

1. *Multiplex PCR - Identification of tem, pse, oxa Genes Responsible for Ampicillin Resistance*

PCR reaction (MIX) was prepared, using a 2 ml Dnase free tube. To the tubes, 45 μl of PCR mix was added, which was briefly vortexed before adding 5 μl of DNA preparation. The tubes were then inserted firmly into the thermal cycler and covered with the lid for DNA amplification. The amplicons were run in a 2% agarose gel, stained with ethidium bromide before viewing and reading the results

| Reagents | Initial concentration | Final concentration | Quantity per sample |
| --- | --- | --- | --- |
| Buffer Taq Gold | 10X | 1X | 5 μl |
| MgCl2 | 25 mM | 2 mM | 4 μl |
| dNTPs | 10 mM | 200 μM | 1 μl |
| Primer *tem* fw (503bp) | 10 μM | 1 μM | 5 μl |
| Primer *tem* rv | 10 μM | 1 μM | 5 μl |
| Primer *pse* fw (419bp) | 10 μM | 1 μM | 5 μl |
| Primer *pse* rv | 10 μM | 1 μM | 5 μl |
| Primer *oxa* fw (708bp) | 10 μM | 1 μM | 5 μl |
| Primer *oxa* rv | 10 μM | 1 μM | 5 μl |
| Taq Gold | 5U/μl | 2.5 U | 0.5 μl |
| H2O | | 4.5 μl | |
| DNA | | 5 μl | |
| Total volume | | 50 | |

*PCR conditions (Thermal profile)*

Pre-denaturation: 95°C 5’;

30 cycles: (95°C 30’’, 55°C 30’’, 72°C 30’’);

Post-extension: 72°C 5’.

*Primers sequence*

| Name | Sequence | Accession number | Reference |
| --- | --- | --- | --- |
| *tem* fw | 5’-TTGGGTGCACGAGTGGGT-3’ | AF126482 | Guerra *et al*., 2001 |
| *tem* rv | 5’-TAATTGTTGCCGGGAAGC-3’ |  |  |
| *pse* fw | 5’-CGCTTCCCGTTAACAAGTAC-3’ | M69058 | Sandvang *et al*., 1997 |
| *pse* rv | 5’-CTGGTTCATTTCAGATAGCG-3’ |  |  |
| *oxa* fw | 5’-AGCAGCGCCAGTGCATCA-3’ | AJ009819 | Guerra *et al*., 2000 |
| *oxa* rv | 5’-ATTCGACCCCAAGTTTCC-3’ |  |  |

The PCR products are visualised by gel electrophoresis in 2% agarose gel and stained with ethidium bromide solution. Observation and images are carried out using a UV light source. The expected amplicons are:

*tem* gene: 503 bp

*pse* gene: 419 bp

*oxa* gene: 708 bp

1. *Multiplex PCR - Identification of tetA, tetB, tetG Genes Responsible for Tetracycline Resistance*

PCR reaction (MIX) was prepared, using a 2 ml Dnase free tube. To the tubes, 45 μl of PCR mix was added, which was briefly vortexed before adding 5 μl of DNA preparation. The tubes were then inserted firmly into the thermal cycler and covered with the lid for DNA amplification. The amplicons were run in a 2% agarose gel, stained with ethidium bromide before viewing and reading the results

| Reagents | Initial concentration | Final concentration | Quantity per sample |
| --- | --- | --- | --- |
| Buffer Taq Gold | 10X | 1X | 5 μl |
| MgCl2 | 25 mM | 2 mM | 4 μl |
| dNTPs | 10 mM | 200 μM | 1 μl |
| Primer *tetA* fw (210bp) | 10 μM | 1 μM | 5 μl |
| Primer *tetA* rv | 10 μM | 1 μM | 5 μl |
| Primer *tetG* fw (500bp) | 10 μM | 1 μM | 5 μl |
| Primer *tetG* rv | 10 μM | 1 μM | 5 μl |
| Primer *tetB* fw (600bp) | 10 μM | 1 μM | 5 μl |
| Primer *tetB* fv | 10 μM | 1 μM | 5 μl |
| Taq Gold | 5U/μl | 2.5 U | 0.5 μl |
| H2O | | 4.5 μl | |
| DNA | | 5 μl | |
| Total volume | | 50 μl | |

*PCR conditions (Thermal profile)*

Pre-denaturation: 95°C 5’;

30 cycles: (95°C 30’’, 55°C 30’’, 72°C 30’’);

Post-extension: 72°C 5’.

*Primers sequence*

| Name | Sequence | Accession number | Reference |
| --- | --- | --- | --- |
| *tetA* fw | 5’-GCTACATCCTGCTTGCCT-3’ | X01367 | Ng *et al*., 1999 |
| *tetA* rv | 5’-CATAGATCGCCGTGAAGA-3’ |  |  |
| *tetB* fw | 5’-TTGGTTAGGGGCAAGTTTTG-3’ | J01830 | Ng *et al*., 1999 |
| *tetB* rv | 5’-GTAATGGGCCAATAACACCG-3’ |  |  |
| *tetG* fw | 5’-GCTCGGTGGTATCTCTGC-3’ | 852437 | Ng *et al*., 1999 |
| *tetG* rv | 5’-AGCAACAGAATCGGGAAC-3’ |  |  |

The PCR products are visualised by gel electrophoresis in 2% agarose gel and stained with ethidium bromide solution. Observation and images are carried out using a UV light source. The expected amplicons are:

 *tetA* gene: 210 bp

 *tetB* gene: 600 bp

 *tetG* gene: 500 bp

*C. Multiplex PCR - Identification of qnrA, qnrB and qnrS Genes Responsible for plasmidic quinolone Resistance*

PCR reaction (MIX) was prepared, using a 2 ml Dnase free tube. To the tubes, 45 μl of PCR mix was added, which was briefly vortexed before adding 5 μl of DNA preparation. The tubes were then inserted firmly into the thermal cycler and covered with the lid for DNA amplification. The amplicons were run in a 2% agarose gel, stained with ethidium bromide before viewing and reading the results

| Reagents | Initial concentration | Final concentration | Quantity per sample |
| --- | --- | --- | --- |
| Buffer Taq Gold | 10X | 1X | 5 μl |
| MgCl2 | 25 mM | 2 mM | 3 μl |
| dNTPs | 10 mM | 200 μM | 1 μl |
| Primer *qnrA* fw (580bp) | 10 μM | 0,2 μM | 5 μl |
| Primer *qnrA* rv | 10 μM | 0,2 μM | 5 μl |
| Primer *qnrB* fw (264bp) | 10 μM | 0,2 μM | 5 μl |
| Primer *qnrB* rv | 10 μM | 0,2 μM | 5 μl |
| Primer *qnrS* fw (428bp) | 10 μM | 0,2 μM | 5 μl |
| Primer *qnrS* fv | 10 μM | 0,2 μM | 5 μl |
| Taq Gold | 5U/μl | 2.5 U | 0.5 μl |
| H2O | | 29,5 μl | |
| DNA | | 5 μl | |
| Total volume | | 50 μl | |

*PCR conditions (Thermal profile)*

Pre-denaturation: 95°C 10’;

35 cycles: (95°C 1’, 54°C 1’, 72°C 1’);

Post-extension: 72°C 10’.

*Primers sequence*

| Name | Sequence | Accession number | Reference |
| --- | --- | --- | --- |
| *qnrA* fw | 5’-TCAGCAAGAGGATTTCTCA-3’ | AY070235 | Wang *et al.,* 2003; Cattoir *et al*., 2007 |
| *qnrA* rv | 5’-GGCAGCACTATTACTCCCA-3’ |  |  |
| *qnrB* fw | 5’-ATGACGCCATTACTGTATTA-3’ |  | Jacoby *et al.,* 2006; Cattoir *et al*., 2007 |
| *qnrB* rv | 5’-GATCGCAATGTGTGAAGTTT-3’ |  |  |
| *qnrS* fw | 5’-ACGACATTCGTCAACTGCAA-3’ |  | Gay *et al*., 2006; Cattoir *et al*., 2007 |
| *qnrS* rv | 5’-TAAATTGGCACCCTGTAGGC-3’ |  |  |

The PCR products are visualised by gel electrophoresis in 2% agarose gel and stained with ethidium bromide solution. Observation and images are carried out using a UV light source. The expected amplicons are:

*qnrA* gene: 580 bp

*qnrB* gene: 264 bp

*qnrS* gene: 428 bp

*D. Multiplex PCR - Identification of catA1, cmlA1 and floR Genes Responsible for chloramphenicol Resistance*

PCR reaction (MIX) was prepared, using a 2 ml Dnase free tube. To the tubes, 45 μl of PCR mix was added, which was briefly vortexed before adding 5 μl of DNA preparation. The tubes were then inserted firmly into the thermal cycler and covered with the lid for DNA amplification. The amplicons were run in a 2% agarose gel, stained with ethidium bromide before viewing and reading the results

| Reagents | Initial concentration | Final concentration | Quantity per sample |
| --- | --- | --- | --- |
| Buffer Taq Gold | 10X | 1X | 5 μl |
| MgCl2 | 25 mM | 2 mM | 4 μl |
| dNTPs | 10 mM | 400 μM | 2 μl |
| Primer *catA1* fw (623bp) | 10 μM | 1 μM | 5 μl |
| Primer *catA1* rv | 10 μM | 1 μM | 5 μl |
| Primer *cmlA1* fw (435bp) | 10 μM | 1 μM | 5 μl |
| Primer *cmlA1* rv | 10 μM | 1 μM | 5 μl |
| Primer *floR* fw (868bp) | 10 μM | 1 μM | 5 μl |
| Primer *floR* fv | 10 μM | 1 μM | 5 μl |
| Taq Gold | 5U/μl | 2.5 U | 0.5 μl |
| H2O | | 3.5 μl | |
| DNA | | 5 μl | |
| Total volume | | 50 μl | |

*PCR conditions (Thermal profile)*:

Pre-denaturation: 95°C 5’;

30 cycles: (95°C 30’’, 55°C 30’’, 72°C 40’’)

Post-extension: 72°C 5’.

*Primers sequene*

| Name | Sequence | Accession number | Reference |
| --- | --- | --- | --- |
| *catA1* fw | 5’-CCACCGTTGATATATCCC-3’ | U40780 | Guerra *et al.,* 2001 |
| *catA1* rv | 5’-CCTGCCACTGATCGCAGT-3’ |  |  |
| *cmlA1* fw | 5’-TGTCATTTACGGCATACTCG-3’ | M64550 | Guerra *et al.,* 2001 |
| *cmlA* rv | 5’-ATCAGGCATCCCATTCCCAT-3’ |  |  |
| *floR* fw | 5’-CACGTTGAGCCTCTATAT-3’ | AF071555 | Ng *et al*., 1999 |
| *floR* rv | 5’-ATGCAGAAGTAGAACGCG-3’ |  |  |

The PCR products are visualised by gel electrophoresis in 2% agarose gel and stained with ethidium bromide solution. Observation and images are carried out using a UV light source. The expected amplicons are:

 *catA1* gene: 623 bp

 *cmlA1* gene: 435 bp

 *floR* gene: 868 bp

*E. One-Target PCR - Identification of sul1 Gene Responsible for Sulfamethoxazole Resistance*

PCR reaction (MIX) was prepared, using a 1.5 ml Dnase free tube. To the tubes, 22.5 μl of PCR mix was added, which was briefly vortexed before adding 2.5 μl of DNA preparation. The tubes were then inserted firmly into the thermal cycler and covered with the lid for DNA amplification. The amplicons were run in a 2% agarose gel, stained with ethidium bromide before viewing and reading the results

| Reagents | Initial concentration | Final concentration | Quantity per sample |
| --- | --- | --- | --- |
| Buffer Taq Gold | 10X | 1X | 2,5 μl |
| MgCl2 | 25 mM | 2 mM | 2μl |
| dNTPs | 10 mM | 200 μM | 0,5 μl |
| Primer *sul1* fw (436bp) | 10 μM | 1 μM | 2,5 μl |
| Primer *sul1* rv | 10 μM | 1 μM | 2,5 μl |
| Taq Gold | 5U/μl | 1.5 U | 0,3 μl |
| H2O | | 12.2 μl | |
| DNA | | 2,5 μl | |
| Total volume | | 25 μl | |

*PCR conditions (Thermal profile)*

Pre-denaturation: 95°C 5’;

30 cycles: (95°C 30’’, 65°C 30’’, 72°C 30’’);

Post-extension: 72°C 5’.

*Primer sequence*

| Name | Sequence | Accession number | Reference |
| --- | --- | --- | --- |
| *sul1* fw | 5’-CTTCGATGAGAGCCGGCGGC-3’ | X12869 | Sandvang *et al.,* 1997 |
| *sul1* rv | 5’-GCAAGGCGGAAACCCGCGCC-3’ |  |  |

The PCR products are visualised by gel electrophoresis in 2% agarose gel and stained with ethidium bromide solution. Observation and images are carried out using a UV light source. The expected amplicons is 436

*F. One-Target PCR - Identification of sul2 Gene Responsible for Sulfamethoxazole Resistance*

PCR reaction (MIX) was prepared, using a 1.5 ml Dnase free tube. To the tubes, 22.5 μl of PCR mix was added, which was briefly vortexed before adding 2.5 μl of DNA preparation. The tubes were then inserted firmly into the thermal cycler and covered with the lid for DNA amplification. The amplicons were run in a 2% agarose gel, stained with ethidium bromide before viewing and reading the results

| Reagents | Initial concentration | Final concentration | Quantity per sample |
| --- | --- | --- | --- |
| Buffer Taq Gold | 10X | 1X | 2,5 μl |
| MgCl2 | 25 mM | 2 mM | 2μl |
| dNTPs | 10 mM | 200 μM | 0,5 μl |
| Primer *sul2* fw (707bp) | 10 μM | 1 μM | 2,5 μl |
| Primer *sul2* rv | 10 μM | 1 μM | 2,5 μl |
| Taq Gold | 5U/μl | 1.5 U | 0,3 μl |
| H2O | | 12.2 μl | |
| DNA | | 2,5 μl | |
| Total volume | | 25 μl | |

*PCR conditions (Thermal profile)*

Pre-denaturation: 95°C 5’;

30 cycles: (95°C 30’’, 58°C 30’’, 72°C 30’’);

Post-extension: 72°C 5’.

*Primer sequence*

| Name | Sequence | Accession number | Reference |
| --- | --- | --- | --- |
| *sul2* fw | 5’-TCAACATAACCTCGGACAGT-3’ | M36657 | Chu *et al.,* 2001 |
| *sul2* rv | 5’-GATGAAGTCAGCTCCACCT-3’ |  |  |

The PCR products are visualised by gel electrophoresis in 2% agarose gel and stained with ethidium bromide solution. Observation and images are carried out using a UV light source. The expected amplicons is 707bp

*G. One-Target PCR - Identification of sul3 Gene Responsible for Sulfamethoxazole Resistance*

PCR reaction (MIX) was prepared, using a 1.5 ml Dnase free tube. To the tubes, 22.5 μl of PCR mix was added, which was briefly vortexed before adding 2.5 μl of DNA preparation. The tubes were then inserted firmly into the thermal cycler and covered with the lid for DNA amplification. The amplicons were run in a 2% agarose gel, stained with ethidium bromide before viewing and reading the results

| Reagents | Initial concentration | Final concentration | Quantity per sample |
| --- | --- | --- | --- |
| Buffer Taq Gold | 10X | 1X | 2,5 μl |
| MgCl2 | 25 mM | 2 mM | 2 μl |
| dNTPs | 10 mM | 200 μM | 0,5 μl |
| Primer *sul3* fw (789bp) | 10 μM | 1 μM | 2,5 μl |
| Primer *sul3* rv | 10 μM | 1 μM | 2,5 μl |
| Taq Gold | 5U/μl | 1.5 U | 0,3 μl |
| H2O | | 12.2 μl | |
| DNA | | 2,5 μl | |
| Total volume | | 25 μl | |

*PCR conditions (Thermal profile)*

Pre-denaturation: 95°C 5’;

30 cycles: (95°C 30’’, 51°C 30’’, 72°C 40’’)

Post-extension: 72°C 5’.

*Primer sequence*

| Name | Sequence | Accession number | Reference |
| --- | --- | --- | --- |
| *sul3* fw | 5’-GAGCAAGATTTTTGGAATCG-3’ | M36657 | Chu *et al.,* 2001 |
| *sul3* rv | 5’-CATCTGCAGCTAACCTAGGGCTTTGGA-3’ |  |  |

The PCR products are visualised by gel electrophoresis in 2% agarose gel and stained with ethidium bromide solution. Observation and images are carried out using a UV light source. The expected amplicons is 789

*H One-Target PCR - Identification of gyrA Gene*

PCR reaction (MIX) was prepared, using a 1.5 ml Dnase free tube. To the tubes, 22.5 μl of PCR mix was added, which was briefly vortexed before adding 2.5 μl of DNA preparation. The tubes were then inserted firmly into the thermal cycler and covered with the lid for DNA amplification. The amplicons were run in a 2% agarose gel, stained with ethidium bromide before viewing and reading the results

| Reagents | Initial concentration | Final concentration | Quantity per sample |
| --- | --- | --- | --- |
| Buffer Taq Gold | 10X | 1X | 5 μl |
| MgCl2 | 25 mM | 2 mM | 4 μl |
| dNTPs | 10 mM | 240 μM | 1,2 μl |
| Primer *gyrA* fw (334bp) | 20 μM | 1 μM | 2,5 μl |
| Primer *gyrA* rv | 20 μM | 1 μM | 2,5 μl |
| Taq Gold | 5U/μl | 1,5 U | 0,3 μl |
| H2O | | 29,5 μl | |
| DNA | | 5 μl | |
| Total volume | | 50 μl | |

*PCR conditions (Thermal profile):*

Pre-denaturation: 95°C 5’;

35 cycles: (95°C 30’’, 52°C 30’’, 72°C 30’’);

Post-extension: 72°C 5’.

| Name | Sequence | Accession number | Reference |
| --- | --- | --- | --- |
| *gyrA* fw | 5’-CCAGATGTHCGHGATGG-3’ | U28377 | Guerra *et al.*, 2003 |
| *gyrA* rv | 5’-ACGAAATCAACSGTYTCTTTTTC-3’ |  |  |

*Primer Sequence*

The PCR products are visualised by gel electrophoresis in 2% agarose gel and stained with ethidium bromide solution. Observation and images are carried out using a UV light source. The expected amplicons is 334bp

*I. One-Target PCR - Identification of dfrA5-14 Gene Responsible for Trimethoprim Resistance*

PCR reaction (MIX) was prepared, using a 1.5 ml Dnase free tube. To the tubes, 22.5 μl of PCR mix was added, which was briefly vortexed before adding 2.5 μl of DNA preparation. The tubes were then inserted firmly into the thermal cycler and covered with the lid for DNA amplification. The amplicons were run in a 2% agarose gel, stained with ethidium bromide before viewing and reading the results

| Reagents | Initial concentration | Final concentration | Quantity per sample |
| --- | --- | --- | --- |
| Buffer Taq Gold | 10X | 1X | 5 μl |
| MgCl2 | 25 mM | 2 mM | 4 μl |
| dNTPs | 10 mM | 200 μM | 1 μl |
| Primer *dfrA5-14* fw (379bp) | 10 μM | 1 μM | 5 μl |
| Primer *dfrA5-14* rv | 10 μM | 1 μM | 5 μl |
| Taq Gold | 5U/μl | 2.5 U | 0,5 μl |
| H2O | | 24,5 μl | |
| DNA | | 5 μl | |
| Total volume | | | |

*PCR conditions (Thermal profile)*

Pre-denaturation: 95°C 5’;

30 cycles: (95°C 30’’, 55°C 30’’, 72°C 30’’);

Post-extension: 72°C 5’.

*Primer sequence*

| Name | Sequence | Accession number | Reference |
| --- | --- | --- | --- |
| *dfrrA5-14* fw | 5’-GATTGGTTGCGGTCCA-3’ |  | Frech *et al.*, 2003 |
| *dfrrA5-14* rv | 5’-CTCAAAAACAACTTCGAAGG-3’ |  |  |

The PCR products are visualised by gel electrophoresis in 2% agarose gel and stained with ethidium bromide solution. Observation and images are carried out using a UV light source. The expected amplicons is 379bp

*J. One-Target PCR - Identification of aac(3)-le Gene Responsible for Gentamycin Resistance*

PCR reaction (MIX) was prepared, using a 1.5 ml Dnase free tube. To the tubes, 22.5 μl of PCR mix was added, which was briefly vortexed before adding 2.5 μl of DNA preparation. The tubes were then inserted firmly into the thermal cycler and covered with the lid for DNA amplification. The amplicons were run in a 2% agarose gel, stained with ethidium bromide before viewing and reading the results

| Reagents | Initial concentration | Final concentration | Quantity per sample |
| --- | --- | --- | --- |
| Buffer Taq Gold | 10X | 1X | 5 μl |
| MgCl2 | 25 mM | 2 mM | 4 μl |
| dNTPs | 10 mM | 400 μM | 2 μl |
| Primer *aac(3)-le* fw (358bp) | 10 μM | 1 μM | 5 μl |
| Primer *aac(3)-le* rv | 10 μM | 1 μM | 5 μl |
| Taq Gold | 5U/μl | 2.5 U | 0,5 μl |
| H2O | | 23,5 μl | |
| DNA | | 5 μl | |
| Total volume | | | |

*PCR conditions (Thermal profile):*

Pre-denaturation: 95°C 5’;

30 cycles: (95°C 30’’, 55°C 30’’, 72°C 30’’);

Post-extension: 72°C 5’.

*Primer sequence*

| Name | Sequence | Accession number | Reference |
| --- | --- | --- | --- |
| *aac(3)-le* fw | 5’-GCAACAAGCCGTCATCAA-3’ | AY463797 | B. Aranda unpublished |
| *aac(3)-le* rv | 5’-CTCTGCTCAACCGCAATGTC-3’ |  |  |

The PCR products are visualised by gel electrophoresis in 2% agarose gel and stained with ethidium bromide solution. Observation and images are carried out using a UV light source. The expected amplicons is 358bp

*K One-Target PCR - Identification of pmrA Gene Responsible for colistin Resistance*

PCR reaction (MIX) was prepared, using a 1.5 ml Dnase free tube. To the tubes, 22.5 μl of PCR mix was added, which was briefly vortexed before adding 2.5 μl of DNA preparation. The tubes were then inserted firmly into the thermal cycler and covered with the lid for DNA amplification. The amplicons were run in a 2% agarose gel, stained with ethidium bromide before viewing and reading the results

| Reagents | Initial concentration | Final concentration | Quantity per sample |
| --- | --- | --- | --- |
| Buffer Taq Gold | 10X | 1X | 5 μl |
| MgCl2 | 25 mM | 2 mM | 4 μl |
| dNTPs | 10 mM | 200 μM | 1 μl |
| Primer *pmrA* fw (668bp) | 5 μM | 0,8 μM | 8 μl |
| Primer *pmrA* rv | 5 μM | 0,8 μM | 8 μl |
| Taq Gold | 5U/μl | 2,5 U | 0,5 μl |
| H2O | | 18,5 μl | |
| DNA | | 5 μl | |
| Total volume | | | |

*PCR conditions (Thermal profile):*

Pre-denaturation: 95°C 5’;

30 cycles: (95°C 30’’, 55°C 30’’, 72°C 40’’);

Post-extension: 72°C 7’.

*Primer sequence*

| Name | Sequence | Accession number | Reference |
| --- | --- | --- | --- |
| *pmrA* fw | 5’-CGCGAATTTCGTGCATGATATG-3’ |  | Sun *et al.,* 2009 |
| *pmrA* rv | 5’-ATGTCCCGATGCTCATTTGGC-3’ |  |  |

The PCR products are visualised by gel electrophoresis in 2% agarose gel and stained with ethidium bromide solution. Observation and images are carried out using a UV light source. The expected amplicons is 668bp

*L One-Target PCR - Identification of pmrB Gene Responsible for colistin Resistance*

PCR reaction (MIX) was prepared, using a 1.5 ml Dnase free tube. To the tubes, 22.5 μl of PCR mix was added, which was briefly vortexed before adding 2.5 μl of DNA preparation. The tubes were then inserted firmly into the thermal cycler and covered with the lid for DNA amplification. The amplicons were run in a 2% agarose gel, stained with ethidium bromide before viewing and reading the results

| Reagents | Initial concentration | Final concentration | Quantity per sample |
| --- | --- | --- | --- |
| Buffer Taq Gold | 10X | 1X | 5 μl |
| MgCl2 | 25 mM | 2 mM | 4 μl |
| dNTPs | 10 mM | 400 μM | 2 μl |
| Primer *pmrB* fw (1070bp) | 5 μM | 0,8 μM | 8 μl |
| Primer *pmrB* rv | 5 μM | 0,8 μM | 8 μl |
| Taq Gold | 5U/μl | 2,5 U | 0,5 μl |
| H2O | | 17,5 μl | |
| DNA | | 5 μl | |
| Total volume | | | |

*PCR conditions (Thermal profile):*

Pre-denaturation: 95°C 5’;

30 cycles: (95°C 30’’, 52°C 30’’, 72°C 1’);

Post-extension: 72°C 10’.

*Primer sequence*

| Name | Sequence | Accession number | Reference |
| --- | --- | --- | --- |
| *pmrB* fw | 5’-AGGAAATTCTGGGCGAGCA-3’ |  | Sun *et al.,* 2009 |
| *pmrB* rv | 5’-CGTTTTCAGCGAAGAGCGA-3’ |  |  |

The PCR products are visualised by gel electrophoresis in 2% agarose gel and stained with ethidium bromide solution. Observation and images are carried out using a UV light source. The expected amplicons is 668bp

*M. One-Target PCR - Identification of parC Gene Responsible for quinolone Resistance*

PCR reaction (MIX) was prepared, using a 1.5 ml Dnase free tube. To the tubes, 22.5 μl of PCR mix was added, which was briefly vortexed before adding 2.5 μl of DNA preparation. The tubes were then inserted firmly into the thermal cycler and covered with the lid for DNA amplification. The amplicons were run in a 2% agarose gel, stained with ethidium bromide before viewing and reading the results

| Reagents | Initial concentration | Final concentration | Quantity per sample |
| --- | --- | --- | --- |
| Buffer Taq Gold | 10X | 1X | 5 μl |
| MgCl2 | 25 mM | 1,5 mM | 3 μl |
| dNTPs | 10 mM | 200 μM | 1 μl |
| Primer *parC* fw (270bp) | 5 μM | 0,5 μM | 5 μl |
| Primer *parC* rv | 5 μM | 0,5 μM | 5 μl |
| Taq Gold | 5U/μl | 1 U | 0,2 μl |
| H2O | | 25,8 μl | |
| *DNA | | 5 μl | |
| Total volume | | | |

*PCR conditions (Thermal profile)*

Pre-denaturation: 95°C 5’;

35 cycles: (95°C 30’’, 53°C 30’’, 72°C 30’’ ) ;

Post-extension: 72°C 5’.

*Primers sequence*

| Name | Sequence | Accession number | Reference |
| --- | --- | --- | --- |
| *parC* fw | 5’ -CTATGCGATGTCAGAGCTGG -3’ |  | Eaves *et al*., 2002 |
| *parC* rv | 5’- TAACAGCAGCTCGGCGTATT -3’ |  |  |

The PCR products are visualised by gel electrophoresis in 2% agarose gel and stained with ethidium bromide solution. Observation and images are carried out using a UV light source. The expected amplicons is 270bp
