# Supplementary material for: Assessing the mechanisms of multi-drug resistant non-typhoidal Salmonella (NTS) serovars isolated from layer chicken farms in Nigeria
Source: PLoS One. 2023 Sep 7;18(9):e0290754. doi: 10.1371/journal.pone.0290754 (PMC10484460; doi:10.1371/journal.pone.0290754)
Supplement: S3 File — (DOCX) [file pone.0290754.s003.docx]

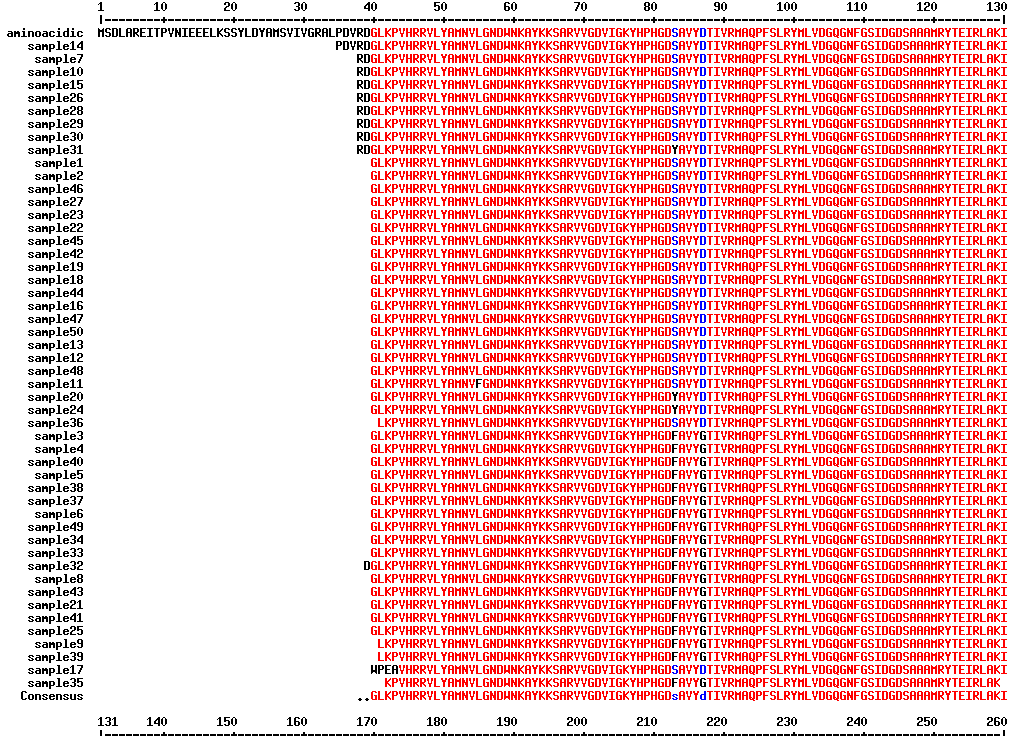


**Figure 1b*:* Amino acid sequence of a portion of *gyrA* gene showing substitution of L for F, S for Y and F, and D for G at positions 55, 83 and 87 respectively. L = Leucine, F = Phenylalanine, S = Serine, Y = Tyrosine, D = Aspartic acid, G = Glycine.**


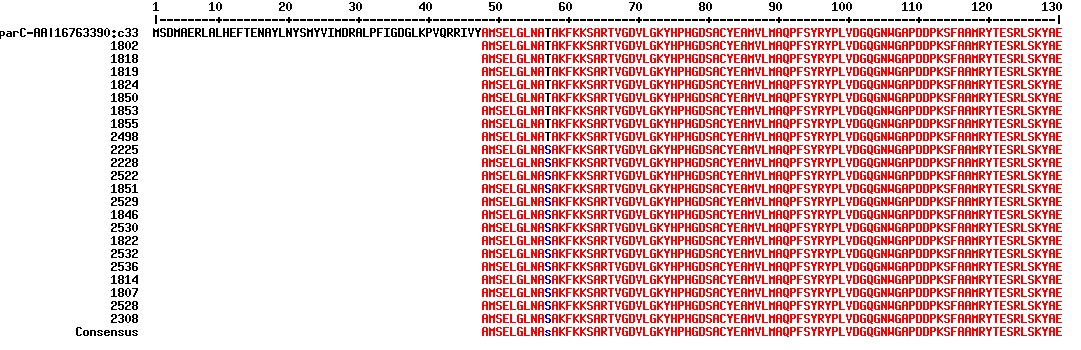


**Figure 1a: Amino acid sequence of a portion of *parC* gene showing substitution of threonine (T) with serine (S) at position 57.**


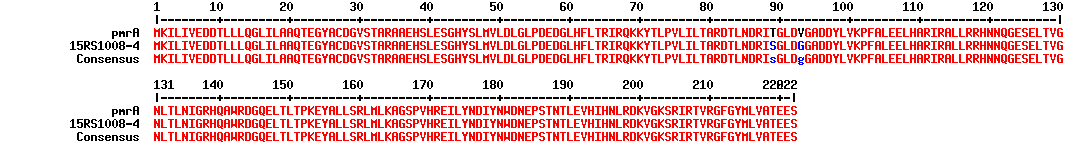


**Figure 1d: Amino acid sequence of a portion of *pmrA* gene showing substitution of threonine (T) with serine (S) and Valine (V) for Glycine (G) at position 89 and 93.**


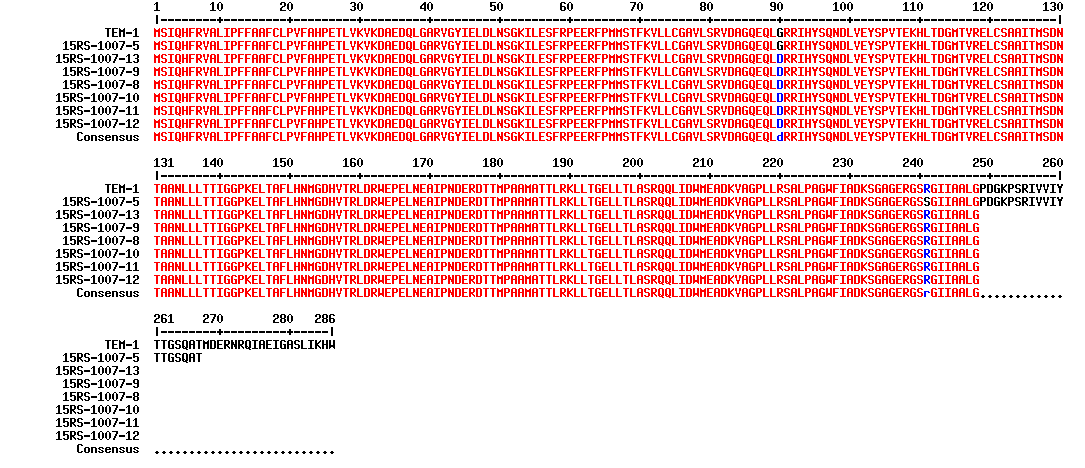


**Figure 1c: Amino acid sequence of a portion of *tem* gene showing substitution of Glycine (G) with Aspartic acid (D) [tem57] and** **Arginine (R) with Serine (S) [tem30] at position 90 and 241 respectively.**
