# Supplementary material for: Assessing the mechanisms of multi-drug resistant non-typhoidal Salmonella (NTS) serovars isolated from layer chicken farms in Nigeria
Source: PLoS One. 2023 Sep 7;18(9):e0290754. doi: 10.1371/journal.pone.0290754 (PMC10484460; doi:10.1371/journal.pone.0290754)
Supplement: S4 File — (DOCX) [file pone.0290754.s004.docx]

**PCR Gel Images**


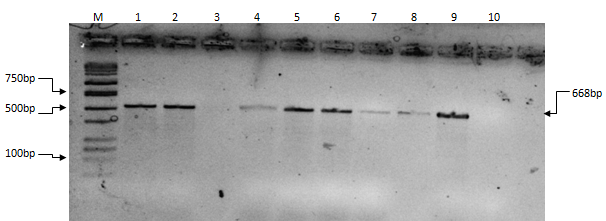


**Figure 1: PCR gel picture for detection of Colistin resistance gene (*pmrA*= 668bp). Lane M is the molecular weight marker, Lanes 1-8 (isolates 1804, 1852, 1845, 1846, 2522, 2528, 2530 and 2536 respectively) are test isolates. Lane 9 is a positive control and lane 10 is the negative control (water).**


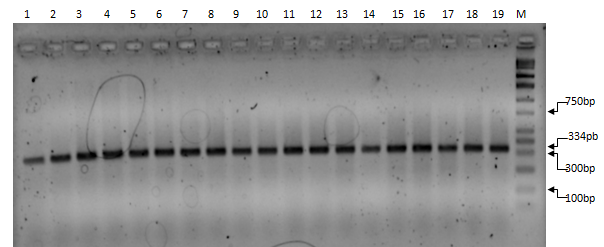


**Figure 2: PCR gel picture for amplification of Quinolone resistance gene (*gyrA*= 334bp). Lane M is the molecular weight marker and lanes 1-18 (isolates 2255, 2258, 2259, 2231, 2238, 2308, 2310, 2311, 1800, 1802, 1807, 1814, 1818, 1819, 1822, 1824, 1835 and 1843 respectively) are test isolates. Lane 19 is the positive control.**


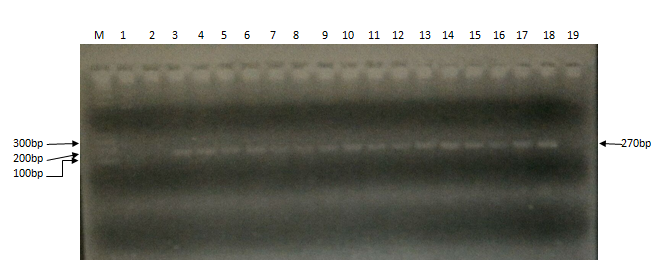


**Figure 3: PCR gel picture for detection of Quinolone resistance gene (*parC*= 270bp). Lane M is the molecular weight marker and lanes 1-17 (isolates 2255, 2258, 2259, 2231, 2238, 2308, 2310, 2311, 1800, 1802, 1807, 1814, 1818, 1819, 1822, 1824 and 1835 respectively) are test isolates. Lane 18 is a positive control and lane 19 is the negative control (water).**


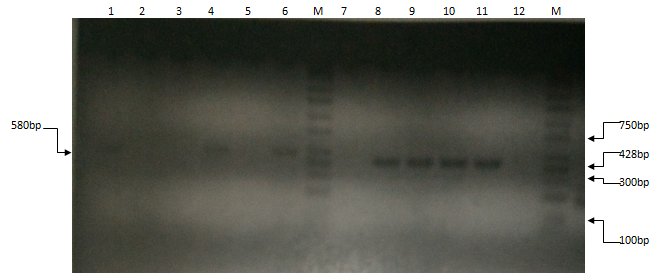


**Figure 4: PCR gel picture for the detection of PMQR genes (*qnrA*=580bp and *qnrS*=428bp). Lane M is the molecular weight marker, lane 1 is *qnrA* positive control, lane 11 is *qnrS* positive control, lanes 2 and 12 are negative controls and lanes 3-10 (isolates 1800, 1802, 1807, 1814, 1818, 1819, 2225 and 1128 respectively) are test isolates.**


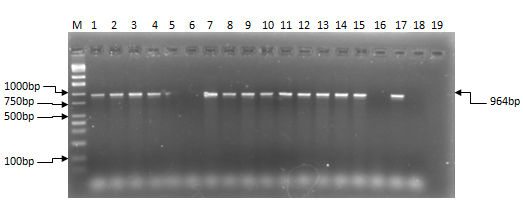


**Figure 5: PCR gel picture for detection of Ampicillin resistance gene (*tem* =964bp). Lane M is the molecular weight marker and lanes 1-16 (isolates 2231, 2238, 2239, 2310, 1800, 1802, 1848, 1851, 2511, 2522, 2524, 2529, 2530, 2739, 2744 and 2528 respectively) are test isolates. Lane 17 is a positive control and lane 18 is the negative control (water).**


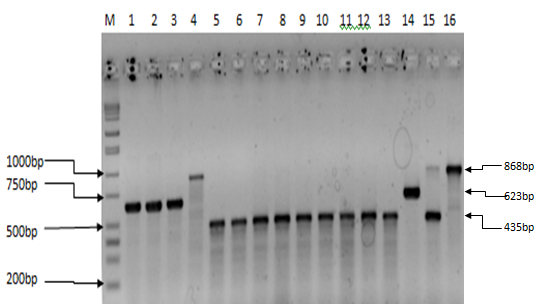


**Figure 6: Multiplex PCR gel picture for amplification of Chloramphenicol resistance genes (*catA1*=623bp, *cmlA1*= 435bp and *floR*= 868bp). Lane M is the molecular weight marker and lanes 1-13 (isolates 1800, 1802, 1851, 2498, 2511, 2522, 2524, 2526, 2529, 2530, 2739, 2744 and 2528 respectively) are test isolates. Lane 14 is *catA1* positive sample, lane 15 is *cmlA1* positive control and lane 16 is *floR* positive control.**


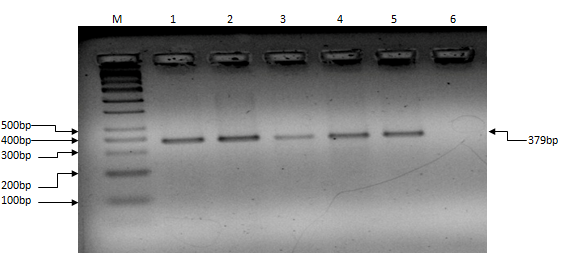


**Figure 7: PCR gel picture for detection of Trimethoprim resistance (*dfrA5-14*=379bp). Lane M is the molecular weight marker and lanes 1-4 (isolates 1800, 1802, 1824 and 1835 respectively) are test isolates. Lane 5 is *dfrA5-14* positive sample and lane 6 is a negative control (water).**


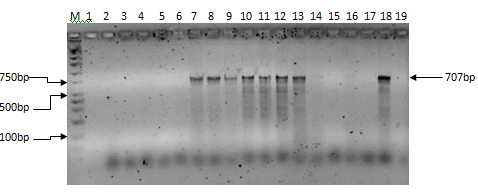


**Figure 8: PCR gel picture for detection of Sulphamethoxazole resistance (*sul 2*= 707bp). Lane M is the molecular weight marker and lanes 1-17 (isolates 2229, 2231, 2238, 2239, 2210, 2311, 1800, 1802, 1807, 1814, 1818, 1822, 1824, 1835, 1843, 1845 and 1847 respectively) are test isolates. Lane 18 is *sul2* positive sample and lane 19 is a negative control (water).**


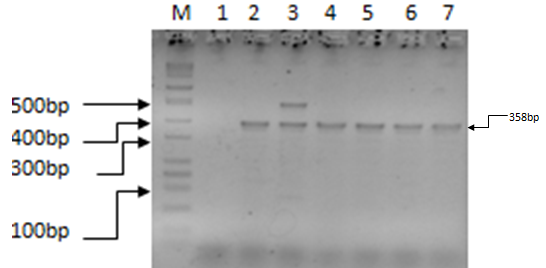


**Figure 9: PCR gel picture for detection of Gentamicin resistance gene (*aac (3)-le* gene = 358bp). Lane M is the molecular weight marker and lane 1 is negative control. Lane 2 is the positive control and lanes 3-7 (isolates 2229, 2231, 2238, 2239 and 2310 respectively) are the test isolates.**


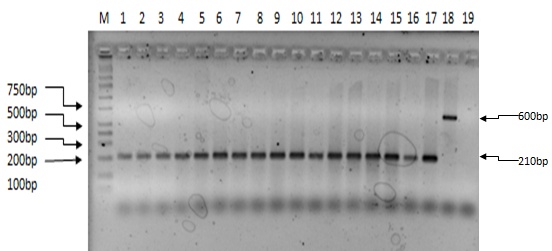


**Figure 10: Multiplex PCR gel picture for the detection of Tetracycline resistance genes (*tetA*=210bp and *tetB*=600bp). Lane M is the molecular weight marker and lanes 1-16 (isolates 2229, 2231, 2238, 2239, 2210, 2311, 1800, 1802, 1807, 1814, 1818, 1822, 1824, 1835, 1843 and 1845) are test isolates. Lane 17 is *tetA* gene positive control, lane 18 is *tetB* gene positive control and lane 19 is a negative control (water).**

**PFGE Gel Images**


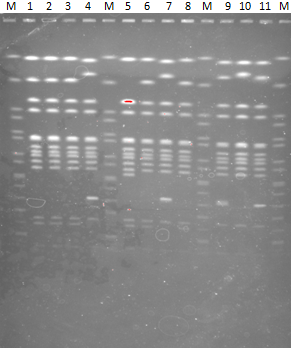


**Figure 11: PFGE gel documentation of *Salmonella* Poona (1,2,3,4,5,6,7,8,9,10,11). *S*. Braenderup (M) was control**


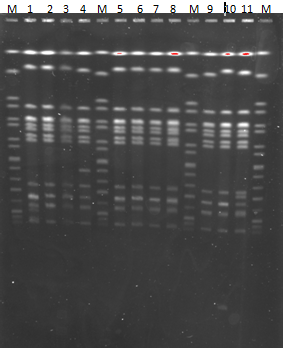


**Figure 12: PFGE gel documentation of *Salmonella* Kentucky (1, 2, 3, 4, 5, 6, 7, 8, 9, 10, 11). *S*. Braenderup (M) was the control.**


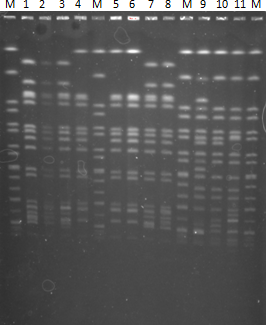


**Figure 13: PFGE gel documentation of *S.* Isangi (1, 2, 3, 4, 5, 6, 7, 8), Larochelle (9, 10 and 11). *S*. Braenderup (M) was the control**


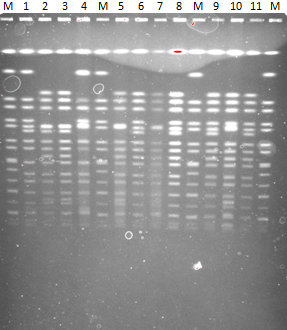


**Figure 14: PFGE gel documentation of *S*. Larochelle** **(1, 2, 3, 4, 5, 6, 7, 8, 9, 10, 11), *S*. Braenderup (M) was the control.**


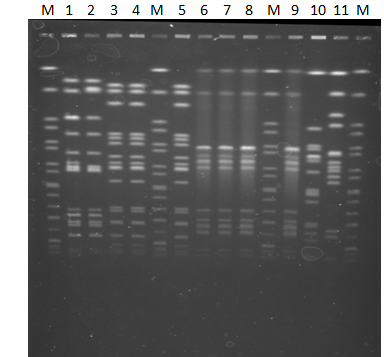


**Figure 15: PFGE gel documentation of *S*. Typhimurium (1, 2, 3, 4, 5), *S*. Nigeria (6, 7, 8, 9, 10) and *S*. Poona (11). *S*. Braenderup (M) was the control**


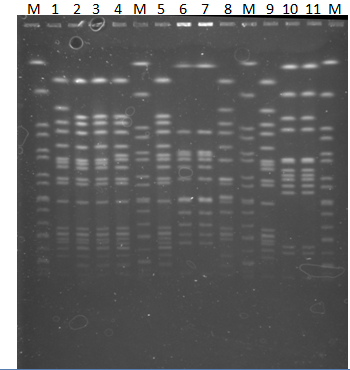


**Figure 16: PFGE gel documentation of *S*. Virchow (1, 2, 3, 4, 5, 6, 7, 8, 9). *S*. Poona (10, 11).*S*. Braenderup (M) was the control**


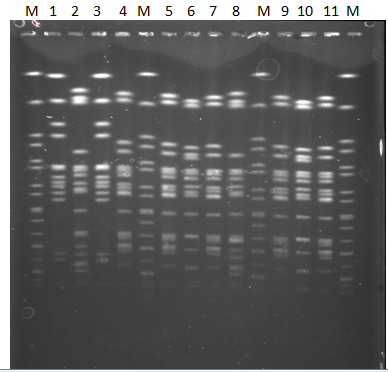


**Figure 17: PFGE gel documentation of *S*. Saintpaul (1, 2, 3, 4, 5, 6, 7, 8, 9). *S*. Poona (10, 11).*S*. Braenderup (M) was the control.**
