# Supplementary material for: Assessing the mechanisms of multi-drug resistant non-typhoidal Salmonella (NTS) serovars isolated from layer chicken farms in Nigeria
Source: PLoS One. 2023 Sep 7;18(9):e0290754. doi: 10.1371/journal.pone.0290754 (PMC10484460; doi:10.1371/journal.pone.0290754)
Supplement: Attachment — Submitted filename: Reviewer comments_Siddiky.docx [file pone.0290754.s005.docx]

**ASSESSING THE MECHANISMS OF MULTI-DRUG RESISTANT NON-TYPHOIDAL SALMONELLA (NTS) SEROVARS FROM LAYER CHICKEN FARMS IN NIGERIA**

**General Comments**

- The manuscript is well articulated and justified
- The work has national and global public health importance
- In introduction, a paragraph on the *Salmonella spp.* as a public health importance is suggested to be incorporated
- There are some anomalies in reference writing in the text. It is suggested to follow the journal style properly
- Following minor correction, the manuscript is considered for publishing in the journal
