# Supplementary material for: Assessing the mechanisms of multi-drug resistant non-typhoidal Salmonella (NTS) serovars isolated from layer chicken farms in Nigeria
Source: PLoS One. 2023 Sep 7;18(9):e0290754. doi: 10.1371/journal.pone.0290754 (PMC10484460; doi:10.1371/journal.pone.0290754)
Supplement: Attachment — Submitted filename: Reviewer Comments.docx [file pone.0290754.s006.docx]

Authors of the manuscript (PONE-D-23-07992) determines the MDR profiles of 200 NTS serovars and determine the mechanisms of resistance by different approaches. The research design and methodologies are acceptable and the data presentation and manuscript write-up are very good. The research findings/results might have significant values in determining AMR control strategies. Authors may consider few points to improve the manuscript further.

Minor Comments

1. Title of the manuscript may be rephrased as below to make it more meaningful:

ASSESSING THE MECHANISMS OF MULTI-DRUG RESISTANCE IN NON-TYPHOIDAL SALMONELLA (NTS) SEROVARS ISOLATED FROM LAYER CHICKEN FARMS IN NIGERIA

2. Authors used 200 isolates of NTS serovars from their previous study and determined their MDR profiles and mechanisms of MDR. Though they referred to their previous work and mentioned the criteria they followed to select 200 isolates, it might be good to include a table (might be a supplementary table) to describe the isolates based on the frequency, serovars, geographical and sample type distribution to help the readers.

3. In conclusion section, authors may describe their key findings shortly within few syntaxes followed by their concluding remarks/ recommendations.
